# Development of a Tablet-Based Outpatient Care Application for People With Dementia: Interview and Workshop Study

**DOI:** 10.2196/59865

**Published:** 2024-12-19

**Authors:** Juliana Supplieth, Sonia Lech, Julie Lorraine O’Sullivan, Robert Spang, Jan‐Niklas Voigt-Antons, Johanna Schuster

**Affiliations:** 1 Institute of Medical Sociology and Rehabilitation Science, Charité–Universitätsmedizin Berlin, corporate member of Freie Universität Berlin, Humboldt-Universität zu Berlin, Charitéplatz 1, Berlin, 10117, Germany, 49 30-450576364; 2 Department of Psychiatry and Psychotherapy, Charité–Universitätsmedizin Berlin, corporate member of Freie Universität Berlin, Humboldt-Universität zu Berlin, Berlin, Germany; 3 Quality and Usability Lab, Faculty IV Electrical Engineering and Computer Science, Technische Universität Berlin, Berlin, Germany; 4 Immersive Reality Lab, Hamm-Lippstadt University of Applied Sciences, Hamm, Germany

**Keywords:** dementia, tablet application development, multidisciplinary health care, feasibility study, general practitioners, digital health care

## Abstract

**Background:**

Dementia management presents a significant challenge for individuals affected by dementia, as well as their families, caregivers, and health care providers. Digital applications may support those living with dementia; however only a few dementia-friendly applications exist.

**Objective:**

This paper emphasizes the necessity of considering multiple perspectives to ensure the high-quality development of supportive health care applications. The findings underscore the importance of incorporating input from stakeholders and the needs of affected families into application development.

**Method:**

A qualitative approach was chosen, consisting of three interviews and an expert workshop. The interviews and the workshop were recorded and transcribed, and qualitative content analysis was carried out according to the methodology described by Kuckartz with the support of MAXQDA.

**Results:**

During the development phases of the application, team meetings and discussions took place. We found that general practitioners and family caregivers play pivotal roles in the treatment and care of people with dementia, often expressing specific preferences and suggestions regarding supportive and assistive technologies. Moreover, the successful development of a useful tablet application requires robust scientific and multidisciplinary discussions and teamwork within the health care community.

**Conclusion:**

This paper underscores the necessity of including multiple scientific, clinical, and technical perspectives to ensure the high-quality development of supportive health care applications. Furthermore, adopting a spiral development approach inclusive of feedback loops is imperative for iterative refinement and enhancement of the application.

## Introduction

### Dementia and Health Care

Dementia impacts approximately 47 million individuals globally, with nearly 10 million new cases emerging each year [1]. In Germany, around 1.6 million people live with dementia [2], posing a significant challenge not only for those affected but also for their families, caregivers, and health care providers. Dementia is characterized as* “*a syndrome resulting from mostly chronic or progressive brain disease, disrupting various higher cortical functions, including memory, cognition, orientation, perception, arithmetic, learning capacity, language, and judgment. Consciousness remains unaffected. Cognitive impairments often coincide with changes in emotional control, social behavior, or motivation...” [3]. These extensive symptoms coincide with a gradual decline in the ability to independently perform daily activities. Consequently, there are physical, psychological, social, and economic effects, leading to disability and dependence among affected individuals [1]. The substantial burden on caregivers, including family, has been widely recognized [1,4,5]. The majority of dementia caregivers are family members of people with dementia, who experience physical and psychological strain due to the declining health status and increasing health needs of the person with dementia [2,6]. For instance, the German College of General Practitioners and Family Physicians [5] provides a brief overview of various health issues faced by caregivers, such as physical health problems (eg, back injuries), mental health issues (eg, depression), and a constant feeling of being overwhelmed. Moreover, previous research has revealed that family caregivers also grapple with challenging behaviors from people with dementia [7].

Information and communication technologies (ICTs) may represent a promising approach to improve dementia treatment, especially for people with dementia that live at home. A systematic review of ICT studies by D’Onofrio et al [8] showed that ICTs can support several daily life activities for people with dementia, allow people with dementia to remain in their own home, increase the quality of life of their caregivers, and decrease health care costs. Evidence-based guidelines and training for general practitioners (GPs) [9] regarding treatment pathways and communication [10], as well as a multiprofessional team approach [11], may represent helpful mechanisms to support GPs.

GPs play a pivotal role in providing health care for people with dementia. Bohlken and Kostev [12] demonstrated that in 2015, GPs treated an average of 29.9 people with dementia, marking a 40% increase compared to 2005 (21.3 people with dementia). However, the role of GPs in diagnosing and treating dementia is subject to debate. Despite their commitment to holistic care and long-term relationships [13], there are significant challenges, including knowledge gaps, reluctance to diagnose, and cost concerns [14], alongside differing care priorities and limitations within the health care system [13]. For instance, in terms of communication, GPs’ decisions to disclose a dementia diagnosis to a patient or their families are influenced by personal beliefs, patient circumstances, systemic care factors, and cultural norms [9]. Additionally, euphemistic terms like “memory problems” are often used instead of medical terminology to describe dementia [9]. Wangler and Jansky [10] observed that GPs’ distant or negative attitudes, as well as their reluctance toward dementia testing and diagnoses, directly impact practical diagnostic outcomes. As a result, GPs may deny responsibility, fear stigmatization of their patients, and avoid confrontations with patients, citing concerns over the effectiveness of interventions, low compensation, and lack of recognition [10]. In summary, existing literature recognizes the indispensable role of GPs in caring for people with dementia and highlights the need to improve primary dementia care.

### Digitization and Technologies

In Germany, current political initiatives aim to expedite the digitalization of the health care system, such as the Digital Act, with the goal of enhancing health care [15]. However, for these efforts to be successful, the technical solutions must align with the needs of the target group. Developing and evaluating assistive technologies necessitates an understanding of the diverse interactions between technology, users, and the contextual environment, as well as knowledge translation [16]. Dugstad et al [17] highlight crucial aspects for the successful implementation of digital technology in dementia care, emphasizing the involvement of key stakeholders from the very beginning, allowing time for exchanges and participation during the initial stages, and advocating for iterative refinement and skill development. Particularly in the development of assistive technologies for individuals with dementia, given their complex circumstances and requirements, extensive collaborative and transdisciplinary engagement is essential, as demonstrated by Boger et al [16].

The research project DemTab (tablet-based outpatient care for people with dementia) aimed to develop and evaluate a tablet-based intervention to enhance treatment for people with dementia in primary care by promoting guideline-based treatment [18,19]. To achieve this goal, a tablet-based intervention was created within the research project. The project was a collaboration between the Institute of Medical Sociology and Rehabilitation Science (ISMR) at Charité–Universitätsmedizin Berlin and the Quality and Usability Lab (QULab) at Technische Universität Berlin. Additionally, a cluster-randomized controlled trial was conducted to investigate the impact of this tablet-based intervention on guideline adherence (primary outcome) and various health-related outcomes for patients and caregivers (secondary outcomes). The tablet-based intervention offers several key functions for people with dementia and their informal caregivers [18]. The final application version contained serious games and included cognitive training games (eg, quizzes), activities focusing on daily living skills, an interactive music program, and a picture gallery for biography work. These tools were designed to engage and stimulate users and enrich their daily lives. Interactive location services provided by the Federal Ministry for Family Affairs, Senior Citizens, Women and Youth highlighted nearby consulting and support services for people with dementia, such as dementia-specific occupational therapy and daycare facilities, as well as resources for informal caregivers like support networks, counseling points, and self-help groups [20]. The communication platform allows for both indirect and direct communication with the GP through health information documentation and messaging, respectively. The care plan developed in collaboration with the GP can be accessed on the tablet by both the person with dementia and their caregivers and provides regular notifications and reminders (eg, for medication intake). Finally, a guided audio-relaxation program, which can be used by both people with dementia and their caregivers, supported relaxation and stress management.

The following functions were designed to support GPs in providing comprehensive, guideline-based care and improving communication with patients and caregivers [18]: (1) a checklist for guideline-based treatment based on the German Dementia Guideline [13]; (2) a prescription support function for antidementia drugs providing guideline-driven treatment recommendations, including automatic reminders for correct medication intake and adjustments; (3) a care development plan that assisted in creating a care plan and determining treatment plans and aims; (4) health information recorded by people with dementia and caregivers displayed on the GPs device, such as general condition or blood pressure values and direct messaging between GPs, people with dementia, and caregivers to enhance communication and action, such as adjusting medication or scheduling appointments; and (5) access to educational material on dementia and dementia care. This also included access for GPs to an electronic version of the German Dementia Guideline [13] and additional information about outpatient dementia care. People with different stages of dementia were included in the study. The application should therefore contain offers for these different stages of the disease. However, the majority (87.9%) of participants in this study had a mild to moderate dementia [19]. For more comprehensive information about the DemTab study, its participants, interventions, and expected outcomes, we refer to the study protocol [18].

### Aim of This Study

This study focused on the initial phase of the DemTab study: the development of a tablet application designed for people with dementia, their caregivers, and GPs in Berlin and the surrounding regions. The objective was to outline the development process of the application within the framework of a feasibility study. The primary aim was to illustrate how health care providers (GPs and experts from supportive organizations in dementia care) contributed to the content development process and how collaborative and interdisciplinary research efforts between the ISMR and the QULab led to the cocreation of a functional health care application. A key objective was to ensure the incorporation of diverse perspectives and professional insights, as well as to facilitate a continuous and iterative research process through feedback loops and team discussions.

## Methods

### Ethical Considerations

Ethical approval was obtained by the ethics committee of the Charité–Universitätsmedizin Berlin (EA1/085/19). Written informed consent was obtained from all participants or legal guardians prior to data collection. All participants had the opportunity to drop out at any time. All data from the interviewees and workshop participants were pseudonymized. The list of reidentifying data was stored separately from the analyzed data, and only authorized individuals have access to them. A data protection concept and a data protection impact assessment were created for the entire project. No compensation was paid for the interviews and the workshop. At the end of the workshop, there was a small buffet lunch for all participants. No one was paid for the pretest application testing in the day clinic or the general practice.

### Research Process and the International Organization for Standardization Standards

Our methodological approach adheres to the International Organization for Standardization (ISO) 9241‐210 standard, titled “Ergonomics of human-system interaction—Part 210: Human-centered design for interactive systems” [21]. The ISO has devised a framework aimed at ensuring systems are both usable and useful, with a focus on users and their needs and requirements, by leveraging human factors, ergonomics, and usability knowledge and techniques [21]. Similarly, the user-centered design (UCD) framework provides a structured methodology for translating ideas into products, prioritizing user preferences and interactions with the final product [22]. The process aims to facilitate natural interactions without altering user behavior or expectations. UCD methodologies are rooted in the ISO 13.180 for ergonomic standards, with the standard 9241‐210 playing a central role in the UCD framework [21]. Our approach to developing the DemTab application aligns with the 4 general phases of the UCD process.

First, we delineated the application’s context, identifying primary user groups and their typical environments based on the method outlined by the International Organization for Standardization [21]. Second, we identified the requirements that our solution must fulfill, encompassing content, usability, and regulatory compliance such as adherence to the General Data Protection Regulation principles [23]. Third, we formulated concepts and prototypes based on the identified requirements, marking the starting point of the design and development phase. Last, we concurrently and continually evaluated our prototype through expert interviews and usability tests with the target user group. This iterative process provided feedback that we could incorporate into subsequent iterations of the development process. Following the process [21], we provided a detailed account of the feedback activities that took place. Additionally, this process exhibits a resemblance to the widely recognized qualitative circular research process, which permits some degree of flexibility and refers back to previous research steps, as it embodies a more dialogical approach [24].

### Process and Data Collection

The DemTab project and the application development process were based on the foundation laid by the previous project PflegeTab. PflegeTab was a tablet-based intervention designed to engage nursing home residents with dementia, with the aim of enhancing quality of life and addressing behavioral symptoms [25]. PflegeTab consisted of various adaptive tablet applications tailored to the participants’ cognitive, functional, and emotional self-regulation abilities. Based on this previous work, the main goal of the DemTab project was to extend this application and create a comprehensive platform for outpatient dementia care. In addition to the existing functionalities for people with dementia, the DemTab application also included additional functionalities developed specifically for GPs and informal caregivers [18,26].

In the initial phase of application development, two GPs from Berlin with experience in dementia primary care were interviewed (step 1): one with expertise in technological approaches in primary care and the other operating in rural areas. Another interview was conducted with a psychologist who worked as a leading manager for the National Dementia Association and was responsible for providing consultations and support to people with dementia and their relatives. These participants were selected based on their valuable expertise and were directly approached by the researchers. A semistructured interview guide was collaboratively developed by the research team. The interviewees were prompted to discuss key aspects of medical and social needs for people with dementia and their informal caregivers, as well as the role of technical support systems in practice. The objective was to gain a deeper understanding of the potential benefits of a tablet-based application for people with dementia, their caregivers, and GPs. The interviews were fully transcribed, and a content analysis was conducted following the methodology outlined by Kuckartz [27], using the MAXQDA 2018 software (VERBI Software) for data analysis.

For the expert workshop (step 1), the researchers extended invitations to two practicing GPs in Berlin, one researcher from the Institute of General Medicine at Charité–Universitätsmedizin Berlin, and two people in managing positions from a support organization based in Berlin that offers voluntary assistance for people with dementia. The workshop was held in September 2018, with participants selected based on their expertise, and they were directly approached by the researchers. While three GPs were initially invited, one was unable to attend. The workshop commenced with a presentation outlining the DemTab study, followed by participants testing version 0.5 of the application and engaging in a focus group discussion. This discussion included a presentation of the results pertaining to the 4 application functions: dementia guidelines, data representation, messaging, and games. Additionally, all workshop attendees completed a questionnaire detailing their personal information and their respective contexts of working with people with dementia and informal caregivers. Following the workshop, members of the research team also completed a brief written survey to capture immediate reflections. The survey comprised of 3 questions including:

- How did you perceive the workshop?
- Which application features or discussion topics were quickly dismissed?
- What topics were extensively discussed?

Team talks and discussions, as well as mutual agreements between researchers from the ISMR and the QULab, were integral (step 2). Biweekly team meetings were held throughout the development period, following a structured procedure. First, the QULab presented new feature additions, then the ISMR researchers tested and evaluated them, then all team members engaged in discussions regarding the user-friendliness of the tool, and finally potential changes and additions informed by insights from interviews and the expert workshop were instituted. The central questions of this study included:

- What valuable knowledge from the previous project PflegeTab should inform our approach?
- What insights from qualitative interviews and workshops should shape specific application functions?
- How can we enhance the implementation of the application in real-world settings?
- What technical features can feasibly be implemented within the timeframe and how?

Additionally, a pretest (step 4) of the DemTab application (version 0.5) was conducted in a geriatric day clinic in Berlin, where people with dementia and staff used the application for 7 days. Furthermore, the application was tested once by a GP who provided feedback and recommendations on content and visualization.

## Results

The main results are presented following the steps explained in this section.

### First Step: Preparation—Interviews and Workshop Findings

#### General Practitioners

Through the interviews and workshop, it became evident that GPs play a crucial role in the comprehensive care of individuals living with dementia and their families. Serving as primary care providers, they are trusted figures who facilitate communication and act as mediators. GP offices also serve as hubs for interdisciplinary and intersectoral communication. It was emphasized that collegial exchanges with specialists and collaboration with family members are vital for ensuring comprehensive home-based care. Moreover, there was a call for increased usage of dementia-specific advisory and support services. One GP highlighted the need for quick access to reliable information, noting the impracticality of consulting extensive guidelines on short notice. Describing the factors influencing care, one GP stated, “Accessing fast, secure information is essential; however, it’s impractical to review all the extensive guidelines on short notice” (Interviewee 1, passage 205).

#### Technology

GPs emphasized the importance of having concise, practical, and filtered information to ensure guideline-oriented treatment and cooperative specialist care. They suggested that data collected by the application should be prioritized and presented graphically to improve comprehension. GPs stressed the significance of choice and self-determination in using the tablet, as well as decisions regarding its functionality. They also advocated for greater integration and networking to facilitate the sharing of information on available assistance and support services. Additionally, there were concerns about the potential for relatives to overload the technical application, particularly with communication functions, which could inadvertently burden GPs. This was discussed critically along with the advantages of video consultations, but they were ultimately rejected by the participating GPs. One GP also commented on potential future technologies stating, “The advantage of video calling is being able to see the person; that adds a different level compared to just talking” (Interviewee 2, passage 130).

#### Relatives and Caregivers

Relatives and caregivers play a significant and multifaceted role in the care of people with dementia at home. Family caregiving occurs under diverse circumstances and can be accompanied by significant stress. Consequently, family members of people with dementia require guidance on medical, psychosocial, and legal matters, as well as access to support services aimed at alleviating stress and fostering social connections tailored to their individual situations and needs. Empowering individuals to help themselves and enhancing their ability to act independently were recognized as crucial objectives. This is illustrated by the following quote stating, “The psychosocial counseling of relatives, aiming to empower them to help themselves and to raise awareness of their own competencies” (Interviewee 8, passage 15). Conclusively, caregivers should be actively engaged in diagnostic and therapeutic processes.

**Figure 1.**
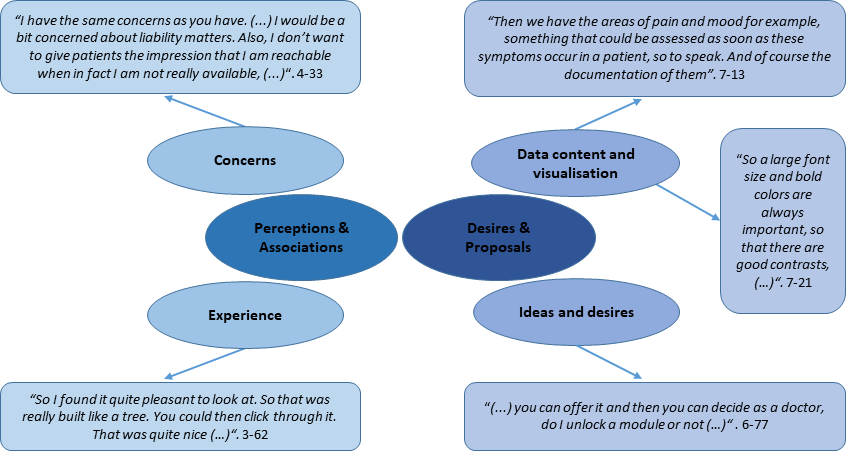
Main categories and subcategories of the workshop with examples. Interviewee ID and passage number for each quote are presented in parentheses, respectively.

The workshop results encompassed information within two primary categories: perceptions and associations, which encompassed experiences and concerns, as well as desires and proposals, which included considerations regarding data content, visualization, and innovative ideas. The principal findings and examples are detailed in Figure 1.

### Second and Third Step: Application Development—Accompanying Team Talks and Discussions

The outcomes of the second step represent the culmination of frequent interdisciplinary team discussions and represent the core of application development. During these team deliberations, in conjunction with the findings of qualitative content analyses, the team concentrated on the study interests and objectives, feasibility, implementation of content, and presentation styles, taking into consideration time and resource constraints. The discussions were marked by their vibrant and emotionally charged nature, especially when deliberating the development of the application’s features. This negotiation of feature development emerged as the primary focus within the team and was carried out in an interdisciplinary manner. For instance, decisions regarding medical content originated from the ISMR, while those concerning technical aspects were made by the QULab.

A substantial portion of the discussions drew upon insights gathered from the interviews and workshop. Based on these insights, the team opted to incorporate ten questions related to the state of health that people with dementia, with or without the assistance of their caregivers, should respond to at least weekly. These questions centered on daily activities such as well-being, hydration, and sleep patterns. Formulated by the IMSR team, these questions aimed to provide GPs with valuable health information and were crafted to be easily accessible by participating individuals. Further, the interdisciplinary discussions with GPs and caregiving experts led to several critical modifications in the application’s design and functionality, including:

- An emergency disclaimer in the chat: A disclaimer was included in the chat function to inform users that the application is not intended for emergencies and that emergency services should be contacted in case of an emergency, with the appropriate emergency number prominently displayed.
- Patient records: The application was updated to include detailed patient profiles, allowing health care providers to access comprehensive patient information quickly and efficiently.
- A single patient view interface: The application’s interface was redesigned to enable a single patient view, where selecting a patient displays all relevant data across various application functions, streamlining the process for health care providers.
- Checklist integration: Based on medical guidelines for dementia care, a checklist feature was added. This checklist allows items to be checked off as completed, with the system automatically resetting the item after three months to ensure regular follow-ups as per the guidelines.
- User interface enhancements: Various suggestions were incorporated to improve the application’s usability and accessibility, ensuring it was intuitive and user-friendly for all health care providers.

### Fourth Step: Application Testing—Feedback and Finalization

The pretest occurred in a geriatric day clinic, involving people with dementia, a social worker, physicians, and one GP who had previously participated in interviews. They provided comprehensive feedback on individual subapplications, focusing on aspects such as user-friendliness, effects on people with dementia and themselves, identification of missing or redundant content, and suggestions for useful additions. Given the time constraints of the project schedule and limited financial resources, discussions on prioritization and timelines were impactful. Throughout the development process, there was a consistent emphasis on ensuring good usability and an appealing presentation style.

## Discussion

The findings of this study indicate that GPs and relatives bear the primary responsibility for the treatment and care of people with dementia, and they harbor specific desires and proposals concerning supportive and assistive technologies. Interdisciplinary discussions are essential to address these needs and develop a practical tablet application. The primary objective of this study was to delineate a multidisciplinary and interdisciplinary approach to the development of an application aimed at enhancing primary care for people with dementia and their caregivers. People with dementia and their caregivers should be supported in their daily lives and disease management, and GPs should be provided with support for their health care. The application should support the joint and interlocking care of those involved.

In conclusion, this study yields various results and implications. First, the study highlights the significant contribution of individuals with diverse disciplinary and professional backgrounds in the development of supportive technical health care applications. In addition to researchers, professionals working in different areas of dementia care provided invaluable insights into care aspects for people with dementia and their family caregivers. These experts should be integrated into the development process as specialists, as they possess implicit knowledge and contributory expertise [28], and experts in local knowledge should be included to leverage the potentials and mitigate the risks of such a study [29]. It is essential for potential users and beneficiaries of the application to be involved from the outset of the development process [21].

We chose to interview employees of the Alzheimer’s Association instead of individuals with dementia and their caregivers. While this approach does not replace the deep, individual perceptions and experiences of affected individuals and their caregivers, it allowed us to gather consolidated information and comprehensive insights accumulated over years of work. Moreover, factors such as time constraints, personal resources, and the challenges of accessing interviewees influenced this decision. While much is known about the progressive and multifaceted challenges and needs of people with dementia, the health care system, professional care institutions, and society still fall short in providing full-time emotional and practical support for them, their caregivers, and their GPs [1]. Additionally, there remains ambiguity surrounding technical solutions [8]. The participation of GPs and employees from the Alzheimer’s Association provided practical insights from health care providers engaged in daily interactions with people with dementia and their caregivers. They shared professional experiences and highlighted daily challenges and needs for improvement. This collaborative process can enhance the acceptance and usage of the application [21].

Our interdisciplinary approach not only facilitated a comprehensive understanding of the needs of GPs and caregivers but also directly influenced the application’s development. The inclusion of features such as the emergency button, patient records, and a checklist system were direct results of feedback from health care professionals. These features were critical in ensuring that the application provided practical, guideline-oriented support in real-world health care settings. Moreover, the interface redesign to include a single patient view and other usability improvements underscored the importance of integrating diverse perspectives to create a tool that is both functional and user-friendly.

Second, the process of developing a health application necessitated multidisciplinary and interdisciplinary teamwork. It is crucial to differentiate between intermittent multidisciplinary teamwork, characterized by juxtaposed disciplinary work, and interdisciplinary teamwork, which entails proactive and interactive collaboration [30]. However, both multidisciplinary and interdisciplinary teamwork are circular scientific endeavors in social care research and involve a process of social negotiation. Effective collaboration involves not only managing coworking due to separate work locations, formal team meetings, and the exchange of information and data but also engaging in discussions and informal team meetings to address preferences, relevancies, and disagreements. Given that each scientific discipline possesses its own specific domain, concepts, and methodologies, multiple constraints and barriers between different disciplines must be dismantled and integrated [31]. Successful multidisciplinary and interdisciplinary teamwork necessitates openness to the knowledge, structures, and practices of other disciplines [32] while consistently advocating for one’s own discipline. Continuous communication and compromise are essential requirements for effective collaboration [21].

Third, future generations are expected to rely more heavily on technical applications for various aspects of life, including health care [33]. Therefore, it is crucial to examine and delve into the development of technical tools, even though the current older generation may not be as technologically savvy as today’s adults and youth. However, it is anticipated that the usage and acceptance of digital technologies will grow in the future [33].

Overall, this study underscores the necessity of multiple scientific, clinical, and technical perspectives to ensure the high-quality development of supportive health care applications. The content and design of such applications for GPs, people with dementia, and their caregivers must be informed by the expertise and experiences of various professional disciplines involved in health care provisions and the usage of such applications in real health care settings. Therefore, multidisciplinary and interdisciplinary collaboration is indispensable in the realm of health care–supporting applications. The development of this dementia care application exemplifies the importance of an interdisciplinary approach in health care technology. The modifications made based on feedback from GPs and other caregiving experts, such as the emergency button, patient profiles, and the checklist feature, highlight how diverse professional insights can lead to a more effective and user-centered design. These changes not only improved the application’s functionality but also enhanced its acceptance and usability among health care providers. The overarching goal is to systematically improve health care for those affected and those involved in their care. Everyday life and care for people with dementia should be supported. Medical disciplines, social sciences, technical expertise, and supporting organizations are all essential to integrate relevant knowledge and competencies into the design and development of useful products, as well as facilitating daily use and active support for health care needs. Furthermore, such development processes require a spiral approach and feedback loops, as well as critical professional and emotional discussions, to reach a consensus and make informed decisions.

## Supplementary material

10.2196/59865 Multimedia Appendix 1 Chat GPT transcript.

## Abbreviations

GP: general practitioner
ICT: information and communication technology
ISMR: Institute of Medical Sociology and Rehabilitation Science
ISO: International Organization for Standardization
QULab: Quality and Usability Lab
UCD: user-centered design

## References

[1] (2017). Global action plan on the public health response to dementia 2017 - 2025. World Health Organization.

[2] (2019). Dementia in europe yearbook 2019: estimating the prevalence of dementia in europe. Alzheimer Europe.

[3] F00-F09 Organische, einschließlich symptomatischer psychischer Störungen [Article in German]. ICD-Code.

[4] (2000). Informationsblatt 7: Die Entlastung pflegender Angehöriger [Article in German]. Deutsche Alzheimer Gesellschaft eV Selbsthilfe Demenz.

[5] Deutsche Gesellschaft für Allgemeinmedizin und Familienmedizin e.V (2018). Pflegende Angehörige von Erwachsenen S3-Leitlinie AWMF-Register-Nr 053-006 DEGAM-Leitlinie Nr 6.

[6] Zwingmann I, Hoffmann W, Michalowsky B (2018). Offene Versorgungsbedarfe pflegender Angehöriger von Menschen mit Demenz. Nervenarzt.

[7] Eggert S, Schnapp P, Sulmann D (2018). Aggressionen und Gewalt in der Pflege. Zentrum für Qualität in der Pflege.

[8] D’Onofrio G, Sancarlo D, Ricciardi F (2017). Information and communication technologies for the activities of daily living in older patients with dementia: a systematic review. J Alzheimers Dis.

[9] Low LF, McGrath M, Swaffer K, Brodaty H (2019). Communicating a diagnosis of dementia: a systematic mixed studies review of attitudes and practices of health practitioners. Dem (Lond).

[10] Wangler J, Jansky M (2020). Dementia diagnostics in general practitioner care. Wien Med Wochenschr.

[11] Pentzek M, Vollmar HC, Wilm S, Leve V (2017). Putting dementia awareness into general practice: the CADIF approach. Z Gerontol Geriatr.

[12] Bohlken J, Kostev K (2018). Diagnostic and prescription behavior of general practitioners and specialist physicians in patients with dementia in 2005 and 2015 in Germany. Psychiatr Prax.

[13] Deutsche Gesellschaft für Psychiatrie und Psychotherapie, Psychosomatik und Nervenheilkunde e. V. (DGPPN), Deutsche Gesellschaft für Neurologie e. V. (DGN) (2023). S3-Leitlinie Demenzen Langfassung.

[14] Deutsche Gesellschaft für Allgemeinmedizin und Familienmedizin e.V (2008). DEGAM-Leitlinie Nr12: Demenz.

[15] (2023). Digitalisation in healthcare. Federal Ministry of Health.

[16] Boger J, Jackson P, Mulvenna M (2017). Principles for fostering the transdisciplinary development of assistive technologies. Disabil Rehabil Assist Technol.

[17] Dugstad J, Eide T, Nilsen ER, Eide H (2019). Towards successful digital transformation through co-creation: a longitudinal study of a four-year implementation of digital monitoring technology in residential care for persons with dementia. BMC Health Serv Res.

[18] Lech S, O’Sullivan JL, Gellert P, Voigt-Antons JN, Greinacher R, Nordheim J (2019). Tablet-based outpatient care for people with dementia. GeroPsych (Bern).

[19] Lech S, O’Sullivan JL, Drewelies J (2021). Dementia care and the role of guideline adherence in primary care: cross-sectional findings from the DemTab study. BMC Geriatr.

[20] (2024). Wegweiser Demenz.

[21] (2019). Ergonomics of human-system interaction — part 210: human-centred design for interactive systems. International Organization for Standardization.

[22] Abras C, Maloney-Krichmar D, Preece J, Bainbridge WS (2004). Encyclopedia of Human-Computer Interaction.

[23] Principles of the GDPR. European Commission.

[24] Witt H (2001). Strategies in qualitative and quantitative research forum qualitative sozialforschung/forum: qualitative social research. Forum Qualitative Sozialforschung.

[25] O’Sullivan JL, Lech S, Gellert P (2022). A tablet-based intervention for activating nursing home residents with dementia: results from a cluster-randomized controlled trial. Int Psychogeriatr.

[26] Lech S, O’Sullivan JL, Wellmann L (2021). Recruiting general practitioners and patients with dementia into a cluster randomised controlled trial: strategies, barriers and facilitators. BMC Med Res Methodol.

[27] Kuckartz U (2014). Qualitative Inhaltsanalyse: Methoden, Praxis, Computerunterstützung 2, Durchges Aufl.

[28] Collins H, Evans R (2007). Rethinking Expertise.

[29] Barrotta P, Montuschi E (2018). Expertise, relevance and types of knowledge. Soc Epistemol.

[30] Klein JT (2010). The Oxford Handbook of Interdisciplinarity.

[31] MacLeod M (2018). What makes interdisciplinarity difficult? Some consequences of domain specificity in interdisciplinary practice. Synthese.

[32] Woiwode H, Froese A (2021). Two hearts beating in a research centers’ chest: how scholars in interdisciplinary research settings cope with monodisciplinary deep structures. Stud Higher Educ.

[33] (2020). Achter Altersbericht: Ältere Menschen und Digitalisierung. Bundesministerium für Familie, Senioren, Frauen und Jugend.

