# Supplementary material for: Development of a Tablet-Based Outpatient Care Application for People With Dementia: Interview and Workshop Study
Source: JMIR Hum Factors. 2024 Dec 19;11:e59865. doi: 10.2196/59865 (PMC11683740; doi:10.2196/59865)
Supplement: Multimedia Appendix 1 [file humanfactors-v11-e59865-s001.docx]

Please improve english

ChatGPT

**ChatGPT**

Of course! Could you please provide some context or specific text that you'd like me to help improve?


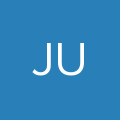


**You**

Dementia represents a major challenge for affected people, their families, caregivers, and healthcare providers. This paper presents the process of the development and feasibility study of a tablet-application. The knowledge of general practitioners, stakeholders and the needs of the affected families are necessary information to develop a supportive healthcare app. GP´s and relatives are mainly responsible for the treatment and care of PwD. They have special desires and proposals towards supporting assistive technologies. At the same time scientific multidisciplinary discussions and teamwork for health care app development are required to meet these needs and to develop a useful tablet-application. This paper shows that multiple perspectives are necessary and urgently needed to ensure high quality development of supportive healthcare apps. Medical disciplines, social and technical science and of course supporting organizations are required to tie relevant knowledge and competences into the design and development of useful products, for daily use and active support for healthcare belongings of the final users. Further a spiral development approach inclusive feedback loops are necessary.

ChatGPT

**ChatGPT**

Dementia presents a significant challenge for individuals affected by it, as well as their families, caregivers, and healthcare providers. This paper outlines the process of developing and assessing the feasibility of a tablet application aimed at addressing this challenge. It underscores the importance of incorporating input from general practitioners, stakeholders, and the needs of affected families to create a supportive healthcare app. General practitioners and relatives play pivotal roles in the treatment and care of Persons with Dementia (PwD), often expressing specific preferences and suggestions regarding supportive assistive technologies. Moreover, successful development of a useful tablet application requires robust scientific, multidisciplinary discussions, and teamwork within the healthcare community. This paper emphasizes the necessity of considering multiple perspectives to ensure the high-quality development of supportive healthcare apps. Collaboration among medical disciplines, social and technical sciences, as well as supporting organizations, is essential to integrate relevant knowledge and expertise into the design and development of practical products tailored to the daily needs and active support for healthcare of end users. Furthermore, adopting a spiral development approach inclusive of feedback loops is imperative for iterative refinement and enhancement of the application.


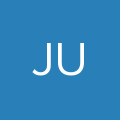


**You**

dementia; tablet-app-development; multidisciplinary health care; feasibility study; general practitioners

ChatGPT

**ChatGPT**

Certainly! Here's a revised version focusing on those key terms:

Addressing dementia presents a formidable challenge, necessitating innovative solutions such as tablet application development. This paper delves into the intricacies of creating and assessing the feasibility of such applications within a multidisciplinary healthcare framework. With a primary focus on general practitioners, the cornerstone of dementia care, and their critical role in treatment and support, this study highlights the importance of soliciting input from stakeholders and affected families. Their insights are invaluable in tailoring supportive assistive technologies to meet specific needs and preferences. Moreover, the collaborative efforts of medical, social, and technical disciplines are indispensable in crafting effective tablet applications. This interdisciplinary approach ensures that diverse perspectives are considered, leading to the development of high-quality healthcare tools. Through iterative refinement and feedback loops, the spiral development methodology employed in this study fosters continuous improvement, ultimately enhancing the usability and efficacy of tablet applications for dementia care.


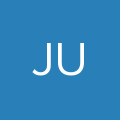


**You**

onnly correct single words, no text construction

ChatGPT

**ChatGPT**

Sure, here are the corrected single words:

dementia; tablet-app development; multidisciplinary healthcare; feasibility study; general practitioners


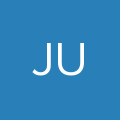


**You**

please improve english

ChatGPT

**ChatGPT**

Certainly! Here's a refined version:

Dementia poses a significant challenge for those affected, their families, caregivers, and healthcare providers. This paper outlines the process of developing and testing the feasibility of a tablet application. Insights from general practitioners, stakeholders, and affected families are crucial for creating a supportive healthcare app. General practitioners and relatives play key roles in the treatment and care of Persons with Dementia (PwD) and offer valuable input on assistive technologies. Successful development requires robust scientific, multidisciplinary discussions, and teamwork within the healthcare community. Collaboration among medical disciplines, social and technical sciences, and supporting organizations is essential to integrate relevant knowledge into the design and development of practical products. Adopting a spiral development approach with feedback loops is crucial for iterative refinement of the application.


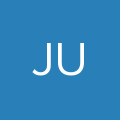


**You**

Dementia affects about 47 million people worldwide, a prevalence that increases with almost 10 million new cases every year (1). In Germany, about 1.6 million people live with dementia (PwD) (2) which represents a major challenge not only for affected individuals, but also for their families, caregivers, and health care providers. Dementia is defined as “a syndrome as a result of a mostly chronic or progressive disease of the brain with disruption of many higher cortical functions, including memory, thinking, orientation, perception, arithmetic, learning ca-pacity, language, and judgment. Consciousness is not clouded. Cognitive impairments are usually accompanied by changes in emotional control, social behavior, or motivation (…).” (3). These comprehensive symptoms go hand in hand with an increasing loss of the ability to perform daily activities independently. Consequently, there are physical, psychological, and social as well as economic impacts, which cause disability and dependency among affected people (1). The special burden on (family) caregivers has been acknowledged numerous times (1, 4, 5). Most dementia caregivers are family members of PwD, who are physically and psychologically affected by the health status and the changing health needs of PwD (2, 6). For example, the German College of General Practitioners and Family Physicians (5) gives a brief overview about diverse health problems, like physical health problems (i.e., back injuries), mental health problems (i.e., depressions) and subjective feeling of being always under request. Further, past research has found that family caregivers are also affected by violent behavior of PwD (7).

ChatGPT

**ChatGPT**

Dementia impacts approximately 47 million individuals globally, with nearly 10 million new cases emerging each year (1). In Germany, around 1.6 million people live with dementia (PwD) (2), posing a significant challenge not only for those affected but also for their families, caregivers, and healthcare providers. Dementia is characterized as "a syndrome resulting from mostly chronic or progressive brain disease, disrupting various higher cortical functions, including memory, cognition, orientation, perception, arithmetic, learning capacity, language, and judgment. Consciousness remains unaffected. Cognitive impairments often coincide with changes in emotional control, social behavior, or motivation..." (3). These extensive symptoms coincide with a gradual decline in the ability to independently perform daily activities. Consequently, there are physical, psychological, social, and economic repercussions, leading to disability and dependence among affected individuals (1). The substantial burden on (family) caregivers has been widely recognized (1, 4, 5). The majority of dementia caregivers are family members of PwD, who experience physical and psychological strain due to the health status and evolving health needs of PwD (2, 6). For instance, the German College of General Practitioners and Family Physicians (5) provides a brief overview of various health issues faced by caregivers, such as physical health problems (e.g., back injuries), mental health issues (e.g., depression), and a constant feeling of being overwhelmed. Moreover, previous research has revealed that family caregivers also grapple with violent behavior from PwD (7).


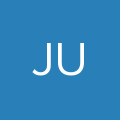


**You**

General practitioners (GPs) play a key role in the health care provision of PwD. Bohlken and Kostev (8) showed that in 2015, GPs treat on average, 29.9 PwD, which represents an in-crease of about 40 % compared to 2005 (21.3 PwD). However, the role of GP´s in diagnostic and therapy of PwD is in a controversial state. Besides the holistic care or trusted long (9), there are also critical elements, like knowledge deficit and renunciation of diagnosis as well as cost reasons (10), different care priorities or just different conditions and possibilities in the medical care area (9). For example, in the field of communication, GPs decision to diagnose and communicate the diagnosis of dementia toward PwD or the family is influenced by per-sonal beliefs and confidences regarding diagnoses and treatment, patient´s circumstances as well as systematic care system factors and cultural norms (11) Further, euphemistic terms such as ‘memory problems’ are more often used than medical terms to describe dementia (ibid.). Wangler and Jansky (12) stated that distance or negative attitude and a critical or re-served standpoint toward dementia tests and diagnoses correlate directly with the practical diagnostic of dementia. Therefore, GPs tend to deny responsibility, fear stigmatization and confrontation with the patients and complain about the lack of application and efficacy as well as a low remuneration and recognition (ibid.). To sum up, previous literature has acknowl-edged the key role of GPs in the provision of care for PwD and a need for improvement in the primary care of PwD.

ChatGPT

**ChatGPT**

General practitioners (GPs) play a pivotal role in providing healthcare for Persons with Dementia (PwD). Bohlken and Kostev (8) demonstrated that in 2015, GPs, on average, treated 29.9 PwD, marking a 40% increase compared to 2005 (21.3 PwD). However, the role of GPs in diagnosing and treating PwD is subject to debate. Despite their commitment to holistic care and long-term relationships (9), there are significant challenges, including knowledge gaps, reluctance to diagnose, and cost concerns (10), alongside differing care priorities and limitations within the healthcare system (9). For instance, in communication, GPs' decisions to disclose a dementia diagnosis to PwD or their families are influenced by personal beliefs, patient circumstances, systemic care factors, and cultural norms (11). Additionally, euphemistic terms like 'memory problems' are often used instead of medical terminology to describe dementia (ibid.). Wangler and Jansky (12) observed that GPs' distance, negative attitudes, and reluctance towards dementia testing and diagnoses directly impact practical diagnostic outcomes. Consequently, GPs may disclaim responsibility, fear stigma, and avoid confrontations with patients, citing concerns over the effectiveness of interventions, low compensation, and lack of recognition (ibid.). In summary, existing literature recognizes the indispensable role of GPs in caring for PwD and underscores the necessity for enhancing primary dementia care.


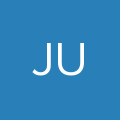


**You**

Besides, there are political efforts to accelerate the digitalization of the healthcare system (like the Digital Act - DigiG) to improve healthcare (15). For this to succeed, however, the technical solution must be suitable for the needs of the target group. The development and evaluation of assistive technologies requires an understanding of different relationships between the technology and people using it and the context where it is used, and translation of knowledge (16). Dugstad, Eide (17) imply important points for successful implementing digital technology in dementia care in practice: the inclusion of key figures from the very beginning, time for exchange and participation in the introductory stage and consider iterative refinement and skill development. Especially, assistive technology development for people with dementia, with their complex situation and needs requires an extensive collaborative and transdisciplinary engagement as Boger, Jackson (16) showed.

ChatGPT

**ChatGPT**

Moreover, there are political initiatives aimed at expediting the digitalization of the healthcare system, such as the Digital Act (DigiG), with the goal of enhancing healthcare (15). However, for these efforts to be successful, the technical solutions must align with the needs of the target demographic. Developing and assessing assistive technologies necessitates an understanding of the diverse interactions between technology, users, and the contextual environment, as well as knowledge translation (16). Dugstad, Eide (17) highlight crucial aspects for the successful implementation of digital technology in dementia care, emphasizing the involvement of key stakeholders from the outset, allowing time for exchange and participation during the initial stages, and advocating for iterative refinement and skill development. Particularly in the development of assistive technologies for individuals with dementia, given their complex circumstances and requirements, extensive collaborative and transdisciplinary engagement is essential, as demonstrated by Boger, Jackson (16).


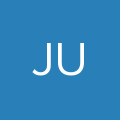


**You**

The research project “DemTab – Tablet-based outpatient care for people with dementia” aimed at developing and evaluating a tablet-based intervention to improve primary care of PwD by fostering guideline-based treatment (18). For this purpose, a tablet-based intervention was developed within the research project. The project partners were the Institute of Medical Sociology and Rehabilitation Science (ISMR) from Charité Universitätsmedizin Berlin, and the Quality and Usability Lab (QULab) from the Technische Universität Berlin. Further, a cluster-randomized controlled trial (cRCT) was conducted to investigate the effects of this tablet-based intervention on guideline adherence (primary outcome) and health-related patient and caregiver outcomes (secondary outcomes). A more detailed description of the DemTab study can be found elsewhere (18).

ChatGPT

**ChatGPT**

The research project "DemTab – Tablet-based outpatient care for people with dementia" aimed to develop and assess a tablet-based intervention to enhance primary care for Persons with Dementia (PwD) by promoting guideline-based treatment (18). To achieve this goal, a tablet-based intervention was created as part of the research project. The project was a collaboration between the Institute of Medical Sociology and Rehabilitation Science (ISMR) at Charité Universitätsmedizin Berlin and the Quality and Usability Lab (QULab) at Technische Universität Berlin. Additionally, a cluster-randomized controlled trial (cRCT) was conducted to examine the impact of this tablet-based intervention on guideline adherence (primary outcome) and various health-related outcomes for patients and caregivers (secondary outcomes). For more comprehensive information about the DemTab study, please refer to the specified source (18).


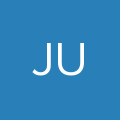


**You**

The present paper focuses on the first step of the DemTab study: the development of a tablet application for PwD, their caregivers and GPs in Berlin and the surrounding area. The aim is to describe the development of the application within the scope of a feasibility study. The key objective is to show how relevant healthcare providers (GPs and experts from supporting or-ganizations in dementia care were involved in the content development of this process and how multi- and interdisciplinary research teamwork between the ISMR and the QULab result-ed in a developed health care app. Key objective however was, to maintain, the integration of multiple perspectives and professions and the single steps of the circular and dialogical re-search process of the technical development through feedback loops and team discussions.

ChatGPT

**ChatGPT**

The present paper focuses on the initial phase of the DemTab study: the development of a tablet application designed for Persons with Dementia (PwD), their caregivers, and General Practitioners (GPs) in Berlin and the surrounding regions. The objective is to outline the process of application development within the framework of a feasibility study. The primary aim is to illustrate how pertinent healthcare providers (GPs and experts from supportive organizations in dementia care) contributed to the content development process, and how collaborative and interdisciplinary research efforts between the ISMR and the QULab led to the creation of a functional healthcare application. A key objective was to ensure the incorporation of diverse perspectives and professional insights, as well as to facilitate a continuous and iterative research process through feedback loops and team discussions.


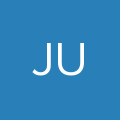


**You**

Our methodological approach is based on the International Organization for Standardization (ISO) Norm ISO 9241-210 Ergonomics of human-system interaction — Part 210: Human-centered design for interactive systems (19). ISO has developed a framework to manage processes and aims to "make systems usable and useful by focusing on the users, their needs and requirements, and by applying human factors/ergonomics, and usability knowledge and techniques" (ibid.,p.vi). Similarly, the User-Centered Design (UCD) frame-work offers a structured methodology for transforming ideas into products by paying attention to what users want and how they can interact with the final product (20). The process should ensure natural interactions without changing behavior or expectations. The UCD methodolo-gies are based on the ISO 13.180 Ergonomics standards, and thus the mentioned standard 9241-210 plays a core role in the UCD framework. The four general phases of the UCD pro-cess capture our approach to developing the DemTab app: First, we specified the app's con-text. We identified primary user groups and their typical environments (compare step 1 in fig-ure 1). Second, we identified requirements our solution must meet, both in terms of content and usability, but also regarding regulations such as General Data Protection Regulation (GDPR) conformance (21). Third, we designed concepts and prototypes based on the identi-fied requirements. This step started the design and development phase. Lastly, but carried out in parallel, we continuously evaluated our product through expert interviews and usability tests with the intended user group. This yielded feedback we could implement in the next iterations of our development process. Figure 1, adapted from the ISO 9241-210 norm, depicts the in-terdependence of human-centered design activities and our process (19).

ChatGPT

**ChatGPT**

Our methodological approach adheres to the International Organization for Standardization (ISO) Norm ISO 9241-210, titled "Ergonomics of human-system interaction — Part 210: Human-centered design for interactive systems" (19). ISO has devised a framework aimed at ensuring systems are both usable and useful, with a focus on users, their needs, and requirements, by leveraging human factors/ergonomics and usability knowledge and techniques (ibid., p.vi). Similarly, the User-Centered Design (UCD) framework provides a structured methodology for translating ideas into products, prioritizing user preferences and interactions with the final product (20). The process aims to facilitate natural interactions without altering user behavior or expectations. UCD methodologies are rooted in the ISO 13.180 Ergonomics standards, with the standard 9241-210 playing a central role in the UCD framework. Our approach to developing the DemTab app aligns with the four general phases of the UCD process:

Firstly, we delineated the app's context, identifying primary user groups and their typical environments (step 1 in Figure 1). Secondly, we identified the requirements our solution must fulfill, encompassing content, usability, and regulatory compliance such as General Data Protection Regulation (GDPR) adherence (21). Thirdly, we formulated concepts and prototypes based on the identified requirements, marking the commencement of the design and development phase. Lastly, conducted concurrently, we continually evaluated our product through expert interviews and usability tests with the target user group. This iterative process provided feedback that we could incorporate into subsequent iterations of our development process. Figure 1, adapted from the ISO 9241-210 norm, illustrates the interdependence of human-centered design activities and our development process (19).


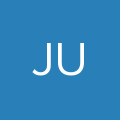


**You**

According to the process, we will give a precise description of the occurred feedback activi-ties. Furthermore, Figure 1 shows similarities with the well-known qualitative circular research process, which allows a partial flexibility and reference back to previous research steps, be-cause it is a more dialogical approach (22).

ChatGPT

**ChatGPT**

Following the process, we will provide a detailed account of the feedback activities that took place. Additionally, Figure 1 exhibits resemblances to the widely recognized qualitative circular research process, which permits some degree of flexibility and refers back to previous research steps, as it embodies a more dialogical approach (22).


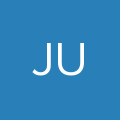


**You**

The present DemTab project and the development process of the application is drawing on a previous project PflegeTab. PflegeTab was a tablet‐based Intervention for activating Nursing Home residents with dementia and aimed to improve Quality of Life and Behavioral Symp-toms in PwD (23). The PflegeTab App comprised several adaptive tablet applications target-ing cognitive and functional abilities as well as emotional self-regulation. Based on this pervi-ous work, the key objective of the DemTab project was to develop an extended application offering a comprehensive platform for the ambulatory care of dementia. Besides including features for PwD, the DemTab application also included additional features designed for GPs and informal caregivers (18, 24).

ChatGPT

**ChatGPT**

The current DemTab project and the application development process build upon the foundation laid by the previous project PflegeTab. PflegeTab was a tablet-based intervention designed to engage nursing home residents with dementia, with the aim of enhancing their Quality of Life and addressing behavioral symptoms (23). The PflegeTab App consisted of various adaptive tablet applications tailored to cognitive, functional, and emotional self-regulation abilities. Leveraging this prior work, the primary objective of the DemTab project was to expand upon this application, creating a comprehensive platform for the outpatient care of dementia. In addition to features for Persons with Dementia (PwD), the DemTab application also incorporated additional functionalities specifically designed for General Practitioners (GPs) and informal caregivers (18, 24).


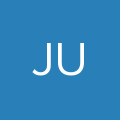


**You**

The first phase of the app development included a qualitative approach comprising three in-terviews and one expert workshop. Two GPs from Berlin (one with expert knowledge on technology approaches in primary care and one, that operates in the rural area, both with ex-pertise in dementia primary care) and one managing director of Alzheimer’s Association Ber-lin, who is responsible for consultations and supportive advice for PwD and their relatives, were interviewed in June and October 2018. All three participants were recruited based on their valuable experiences and contacted directly by the researchers. A half-structured inter-view guideline was sketched by all team members. The interviewees were asked to report on important aspects of general medical and social needs for PwD and their informal caregivers as well as the role of technical support systems in practice. The aim was to gain a better un-derstanding of the potential of a tablet-based application for PwD, their caregivers and GPs. Interviews were completely transcribed, and a content analysis has been carried out, based on, Kuckartz (25) and using the supporting software MAXQDA 2018 (26) for data analyses.

ChatGPT

**ChatGPT**

In the initial phase of app development, a qualitative approach was adopted, consisting of three interviews and one expert workshop. Two General Practitioners (GPs) from Berlin, one with expertise in technology approaches in primary care and the other operating in rural areas, both with experience in dementia primary care, were interviewed alongside one managing director of the Alzheimer’s Association Berlin, responsible for providing consultations and support to Persons with Dementia (PwD) and their relatives. These participants were selected based on their valuable insights and were directly approached by the researchers. A semi-structured interview guide was collaboratively developed by the research team. The interviewees were prompted to discuss key aspects of medical and social needs for PwD and their informal caregivers, as well as the role of technical support systems in practice. The objective was to gain a deeper understanding of the potential benefits of a tablet-based application for PwD, their caregivers, and GPs. The interviews were transcribed in full, and content analysis was conducted following the methodology outlined by Kuckartz (25), utilizing the MAXQDA 2018 software (26) for data analysis.


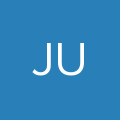


**You**

For the expert-workshop, the researchers invited two GPs practising Berlin, two advisors from a support organization based in Berlin providing voluntary support for PwD and one research-er from the Institute of General Medicine of the Charité - Universitätsmedizin Berlin. The workshop took place in September 2018. Participants were, again, recruited based on their experiences and directly contacted by the researchers. A total of three GPs were invited, one GP was unable to attend. The workshop started with a presentation of the DemTab-project study, followed by a test of the app version 0.5 by participants and a subsequently focused group discussion including a presentation of the results of the four app- functions (dementia-guideline, data representation, messages, and games) and their representation. In addition, all workshop participants filled in a questionnaire about their personal data and their individual context of work with PwD and their informal caregivers. The focus group discussion and the presentation were audio recorded and transliterated. The content analyses were also based on (25) and with support of MaxQDA (26).

ChatGPT

**ChatGPT**

For the expert workshop, the researchers extended invitations to two practicing GPs in Berlin, two advisors from a support organization based in Berlin offering voluntary assistance for Persons with Dementia (PwD), and one researcher from the Institute of General Medicine at Charité - Universitätsmedizin Berlin. The workshop was held in September 2018, with participants selected based on their expertise and directly approached by the researchers. While three GPs were initially invited, one was unable to attend. The workshop commenced with a presentation outlining the DemTab project study, followed by participants testing version 0.5 of the app and engaging in a focused group discussion. This discussion included a presentation of the results pertaining to the four app functions: dementia guideline, data representation, messaging, and games. Additionally, all workshop attendees completed a questionnaire detailing their personal information and their respective contexts of working with PwD and their informal caregivers. Both the focus group discussion and the presentation were audio recorded and transcribed. Content analyses were conducted following the methodology outlined by Kuckartz (25), with support from MaxQDA (26).


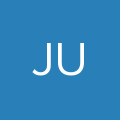


**You**

Besides the workshop- participants, the research team members also filled in a short-written survey directly after the workshop took place. Thereby three questions should be answered, to reflect the workshop immediately: 1) How did you feel about the workshop? 2) Which app or other discussion contents were quickly discarded? and 3) What was discussed at length? The second and third step of the app development (figure 1, step 2) included team talks and discussions, and mutual agreements between the researchers of the ISMR and the QULab. During the development period, bi-weekly team meeting where hold. The procedure was as follows: The QULab presented the new app version, the IMSR researchers checked it and finally all discussed on necessary user-friendliness of the respective tool, possible and neces-sary changes and additions. Further the discussion was based on the results of the interviews and the expert workshop. Central questions have been: Which relevant knowledge from the previous project “PflegeTab” should be considered? Which is the relevant knowledge gained from the qualitative interviews and workshop regarding specific functions of the app? How can we improve the implementation of the application in the field? What can be technically implemented in the timeframe and how? Further, a pretest (figure 1, step 4) of the DemTab-App (version 0.5) was carried out in both, a geriatric day clinic in Berlin where PwD and the staff used the application for seven days, and once with a GP, who used the application and gave comments and recommendations on the content and visualization of the application.

ChatGPT

**ChatGPT**

Following the workshop, members of the research team also completed a brief written survey to capture immediate reflections. The survey comprised three questions: 1) How did you perceive the workshop? 2) Which app features or discussion topics were quickly dismissed? and 3) What topics were extensively discussed?

During the subsequent phases of app development (Figure 1, Step 2), team talks and discussions, as well as mutual agreements between researchers from the ISMR and the QULab, were integral. Bi-weekly team meetings were held throughout the development period, following a structured procedure: The QULab presented the new app version, the ISMR researchers assessed it, and all team members engaged in discussions regarding the user-friendliness of the tool, potential changes and additions, informed by insights from interviews and the expert workshop. Central questions included: What valuable knowledge from the previous project "PflegeTab" should inform our approach? What insights from qualitative interviews and workshops should shape specific app functions? How can we enhance the implementation of the application in real-world settings? What technical features can feasibly be implemented within the timeframe and how? Additionally, a pretest (Figure 1, Step 4) of the DemTab-App (version 0.5) was conducted in a geriatric day clinic in Berlin, where Persons with Dementia (PwD) and staff utilized the application for seven days. Furthermore, the application was tested once by a General Practitioner (GP) who provided feedback and recommendations on content and visualization.


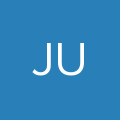


**You**

The principal results are presented using the steps as shown in figure 1.

ChatGPT

**ChatGPT**

Certainly! Here's a presentation of the principal results following the steps shown in Figure 1:

Step 1: Context Specification

- Identified primary user groups: Persons with Dementia (PwD), informal caregivers, and General Practitioners (GPs).
- Characterized typical environments and needs of each user group.

Step 2: Requirement Identification

- Recognized requirements for the application, including content, usability, and regulatory compliance such as General Data Protection Regulation (GDPR) conformance.
- Considered insights from previous project "PflegeTab" and qualitative interviews and workshops.

Step 3: Concept and Prototype Design

- Developed concepts and prototypes based on identified requirements.
- Initiated the design and development phase, focusing on user-friendliness and practicality.

Step 4: Continuous Evaluation

- Conducted a pretest of the DemTab-App (version 0.5) in a geriatric day clinic and with a General Practitioner (GP).
- Gathered feedback and recommendations on content, visualization, and functionality of the application.

Overall, the iterative process involved close collaboration between the ISMR and QULab researchers, integrating feedback from stakeholders and users to refine and improve the DemTab application.


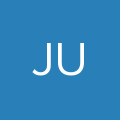


**You**

no text generation, only correct aamd improve english

ChatGPT

**ChatGPT**

Certainly! Here's a refined version:

The principal findings are presented in accordance with the steps outlined in Figure 1.

Step 1: Context Specification

- Identified primary user groups: Persons with Dementia (PwD), informal caregivers, and General Practitioners (GPs).
- Characterized the typical environments and needs of each user group.

Step 2: Requirement Identification

- Recognized the requirements for the application, including content, usability, and regulatory compliance such as General Data Protection Regulation (GDPR) conformance.
- Considered insights from the previous project "PflegeTab" and qualitative interviews and workshops.

Step 3: Concept and Prototype Design

- Developed concepts and prototypes based on the identified requirements.
- Initiated the design and development phase, focusing on user-friendliness and practicality.

Step 4: Continuous Evaluation

- Conducted a pretest of the DemTab-App (version 0.5) in a geriatric day clinic and with a General Practitioner (GP).
- Gathered feedback and recommendations on the content, visualization, and functionality of the application.

Overall, the iterative process involved close collaboration between the ISMR and QULab researchers, integrating feedback from stakeholders and users to refine and enhance the DemTab application.


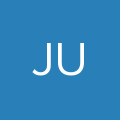


**You**

The three interviews' results can be summarized in three main categories: 1) “General Practitioners” 2) “Technique” and 3) “Relatives and caregivers”. 1) Category “General Practitioners”

ChatGPT

**ChatGPT**

The results of the three interviews can be summarized into three main categories: 1) "General Practitioners", 2) "Technology", and 3) "Relatives and Caregivers".

1. Category "General Practitioners":


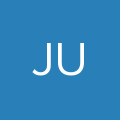


**You**

Category "General Practitioners": First, GPs play a significant role in the comprehensive care of people living with dementia and their relatives. As primary care providers, they are a trusted person and mediator as well as a place for interdisciplinary and sectoral information pooling. Collegial exchange with specialists and cooperation with relatives are necessary to ensure complex domestic care. Contacting and mediating with dementia-specific advisory and support institutions should be more frequent. One GP described the following factors in-fluencing care: “You just need access to fast, safe information and cannot read all the guide-lines. (...) They are just very, very extensive and it is not practical to include them at short notice. “ TN 1- 205

ChatGPT

**ChatGPT**

Under the category "General Practitioners", it was evident that GPs play a crucial role in the comprehensive care of individuals living with dementia and their families. Serving as primary care providers, they are trusted figures who facilitate communication and serve as intermediaries. GPs also act as hubs for interdisciplinary and sectoral information sharing. It was emphasized that collegial exchanges with specialists and collaboration with family members are vital for ensuring comprehensive home-based care. Moreover, there was a call for increased utilization of dementia-specific advisory and support services. One GP highlighted the need for quick access to reliable information, noting the impracticality of consulting extensive guidelines on short notice.


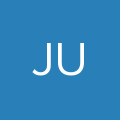


**You**

You just need access to fast, safe information and cannot read all the guidelines. (...) They are just very, very extensive and it is not practical to include them at short notice

ChatGPT

**ChatGPT**

Accessing fast, secure information is essential; however, it's impractical to review all the extensive guidelines on short notice.


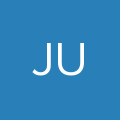


**You**

Second, to ensure a guideline-oriented treatment, the GPs wanted short, practicable and fil-tered information for the GP and cooperative specialist care. Data collected in the app could be prioritized and displayed graphically for easy readability. Choice and self-determination regarding the tablet display and decisions on the use of itself are important for the usage of technology. Greater linking and networking should be sought for a helpful pooling of infor-mation on assistance offers and support. In addition, there were fears of overstretching the technical application (communication function) by relatives, which could increase the burden on general practitioners. Regarding potential future technologies one GP said:

ChatGPT

**ChatGPT**

Secondly, GPs emphasized the importance of having concise, practical, and filtered information to ensure guideline-oriented treatment and cooperative specialist care. They suggested that data collected in the app should be prioritized and presented graphically for easy comprehension. GPs stressed the significance of choice and self-determination in using the tablet, as well as decisions regarding its functionality. They also advocated for greater integration and networking to facilitate the sharing of information on available assistance and support services. Additionally, there were concerns about the potential for relatives to inadvertently overload the technical application, particularly with communication functions, which could inadvertently burden GPs. One GP also commented on potential future technologies:


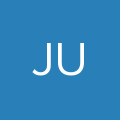


**You**

“(…) the advantage of video calling is to see the person, that's a different level than just talk-ing, (…).”

ChatGPT

**ChatGPT**

"The advantage of video calling is being able to see the person; that adds a different level compared to just talking."


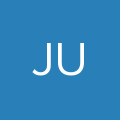


**You**

Third, relatives and caregivers are significantly and comprehensively involved in the care of PwD at home. Family care takes place under a wide variety of conditions and can be associ-ated with stress. Accordingly, family members of PwD need advice on medical, psychoso-cial, and legal issues as well as relieving and social, networking support services according to their personal situations and needs. Helping people to help themselves and strengthening their capacity to act are considered as important goals. Like the following quote demonstrates: “(…) the psychosocial counselling of relatives, that is to say to empower relatives in the sense of helping them to help themselves and to make them aware of their own competenc-es.” TN 8- 15 Concluding caregivers should also be involved in diagnostic and therapeutic processes

ChatGPT

**ChatGPT**

Thirdly, relatives and caregivers play a significant and multifaceted role in the care of Persons with Dementia (PwD) at home. Family caregiving occurs under diverse circumstances and can be accompanied by significant stress. Consequently, family members of PwD require guidance on medical, psychosocial, and legal matters, as well as access to support services aimed at alleviating stress and fostering social connections tailored to their individual situations and needs. Empowering individuals to help themselves and enhancing their ability to act independently are recognized as crucial objectives. As illustrated by the following quote: "(...) the psychosocial counseling of relatives, aiming to empower them to help themselves and to raise awareness of their own competencies." Conclusively, caregivers should be actively engaged in diagnostic and therapeutic processes.


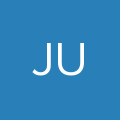


**You**

The workshop-results include information in two main categories: 1) “perceptions and asso-ciations”, like experience and concerns and 2) “desires and proposals”, like data content and their visualization and ideas. The main results and examples are presented in Figure 2.

ChatGPT

**ChatGPT**

The workshop results encompass information within two primary categories:

1. "Perceptions and Associations", which encompass experiences and concerns, and
2. "Desires and Proposals", which include considerations regarding data content, visualization, and innovative ideas. The principal findings and examples are detailed in Figure 2.


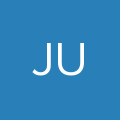


**You**

The second step's results encapsulate the output of the frequent interdisciplinary team talks, which can be considered as the heart of the app development. In these team discussions, along with the results of the qualitative content analyses, the team focused on the study inter-ests and objectives, feasibility and implementation of content, and presentation styles under time and effort factors.

ChatGPT

**ChatGPT**

The outcomes of the second step represent the culmination of frequent interdisciplinary team discussions, which are deemed as the core of app development. During these team deliberations, in conjunction with the findings of qualitative content analyses, the team concentrated on the study interests and objectives, feasibility, and implementation of content, as well as presentation styles, considering time and resource constraints.


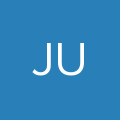


**You**

The discussions were characterized by their lively and emotional nature, particularly when discussing the development of the app's features. This negotiation of feature development became the primary task within the team and was conducted on an interdisciplinary basis. For instance, while decisions about medical content came from the ISMR, decisions about the technical aspects were made by the QULab.

ChatGPT

**ChatGPT**

The discussions were marked by their vibrant and emotionally charged nature, especially when deliberating the development of the app's features. This negotiation of feature development emerged as the primary focus within the team and was carried out in an interdisciplinary manner. For instance, decisions regarding medical content originated from the ISMR, while those concerning technical aspects were made by the QULab.


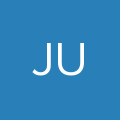


**You**

A significant part of the discussions was based on insights gleaned from interviews and work-shops. For example, the team decided on ten questions related to the "state of health" that should be answered at least weekly from the People with Dementia (PwD) with or without the help of their caring relatives. The questions focused on daily activities, such as wellbeing, drinking, and sleeping. These questions, which were formulated by the IMSR team, provided useful health information for General Practitioners (GPs) and were designed to be easy for people to understand and answer.

ChatGPT

**ChatGPT**

A substantial portion of the discussions drew upon insights gathered from interviews and workshops. For instance, based on these insights, the team opted to incorporate ten questions related to the "state of health" that individuals with dementia (PwD), with or without the assistance of their caregivers, should respond to at least weekly. These questions centered on daily activities such as wellbeing, hydration, and sleep patterns. Formulated by the IMSR team, these questions aimed to furnish General Practitioners (GPs) with valuable health information and were crafted to be easily comprehensible and answerable by individuals.


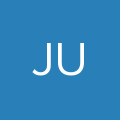


**You**

The pretest took place in a geriatric day clinic with PwD, a social worker and physicians as well as with one GP, who has already been interviewed and took part in the workshop. They gave comprehensive feedback based on the individual subapps regarding user friendliness, effects on PwD, like irritations and themselves, general missing and superfluous content as well as useful additions. Given the constraints of the tight project schedule and limited financial resources, degrees of priority and timelines were also significant topics of discussion. Overall, the drive for good usability and an appealing presentation style were continually present during the development process.

ChatGPT

**ChatGPT**

The pretest occurred in a geriatric day clinic, involving Persons with Dementia (PwD), a social worker, physicians, and one General Practitioner (GP) who had previously participated in interviews and the workshop. They provided comprehensive feedback on individual sub-apps, focusing on aspects such as user-friendliness, effects on PwD and themselves, identification of missing or redundant content, and suggestions for useful additions.

Given the constraints of the tight project schedule and limited financial resources, discussions on prioritization and timelines were significant. Throughout the development process, there was a consistent emphasis on ensuring good usability and an appealing presentation style.


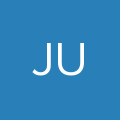


**You**

The results can be summarized as follows: GP´s and relatives are mainly responsible for the treatment and care of PwD. They have special desires and proposals towards supporting as-sistive technologies. Interdisciplinary discussions are needed to meet these needs and to de-velop a useful tablet-application. The key objective of the present paper was to describe the process and the multi- and partial interdisciplinary approach in the app development of an application to improve primary care for PwD and their caregivers. Finally, a variety of results and implications can be drawn from the present paper.

ChatGPT

**ChatGPT**

The findings can be summarized as follows: General Practitioners (GPs) and relatives bear primary responsibility for the treatment and care of Persons with Dementia (PwD). They harbor specific desires and proposals concerning supportive assistive technologies. Interdisciplinary discussions are essential to address these needs and develop a practical tablet application. The primary objective of this paper was to delineate the process and the multi- and partial interdisciplinary approach in the development of an application aimed at enhancing primary care for PwD and their caregivers.

In conclusion, the present paper yields various results and implications.


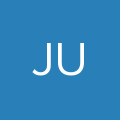


**You**

Firstly, it shows how many people with different disciplinary and professional backgrounds are of immense importance in the app development process for supportive technical healthcare. Along with researchers from different disciplines, members of various professions. working in different fields of everyday dementia care gave substantial and indispensable in-formation on care aspects for PwD and their caring relatives. These experts should be inte-grated in the developing process as specialists with implicit knowledge and contributory ex-pertise (27) and additional as experts of local knowledge to exploit the potentials and risks of such a study project (28). These potential users and beneficiaries of the app should be in-volved from the beginning of the development process (19). We decided to interview employ-ees of Alzheimer Societies Association instead of single PwD and their caring relatives. Of course, this does not replace the profound, individual perception and experience of affected people and their caregivers. However, this enabled us to get bundled information and com-prehensive experience from years of work. Besides, it is a question of time capacity, personal resources and of course the more difficult access to interviewees. Certainly, a lot of is known about the progressive and complex problems and various needs of PwD, but still the healthcare system and professional care institutions as well as the society does not meet the daily fulltime emotional and practical support of PwD, their caregivers and their GP´s (1). Ad-ditionally, technical solutions are not fully clarified (14). Participating GPs and employees from Alzheimer Societies were practical healthcare providers, who are in daily business with PwD and their caregivers. They shared professional experience from their everyday work with us including daily problems and several needs to improve these problems. This process can raise the acceptance and use of the app (19).

ChatGPT

**ChatGPT**

Firstly, the paper highlights the significant contribution of individuals with diverse disciplinary and professional backgrounds in the development of supportive technical healthcare applications. In addition to researchers from various disciplines, professionals working in different domains of dementia care provided invaluable insights into care aspects for Persons with Dementia (PwD) and their caregivers. These experts should be integrated into the development process as specialists possessing implicit knowledge and contributory expertise (27), as well as experts in local knowledge to leverage the potentials and mitigate the risks of such a study project (28). It is essential for potential users and beneficiaries of the application to be involved from the outset of the development process (19).

We chose to interview employees of Alzheimer's Societies Association instead of individual PwD and their caregivers. While this approach does not replace the deep, individual perceptions and experiences of affected individuals and their caregivers, it allowed us to gather consolidated information and comprehensive insights accumulated over years of work. Moreover, factors such as time constraints, personal resources, and the challenges of accessing interviewees influenced this decision. While much is known about the progressive and multifaceted challenges and needs of PwD, the healthcare system, professional care institutions, and society still fall short in providing full-time emotional and practical support for PwD, their caregivers, and their GPs (1). Additionally, there remains ambiguity surrounding technical solutions (14).

The participation of GPs and employees from Alzheimer's Societies provided practical insights from healthcare providers engaged in daily interactions with PwD and their caregivers. They shared professional experiences and highlighted daily challenges and needs for improvement. This collaborative process can enhance the acceptance and utilization of the application (19).

2 / 2


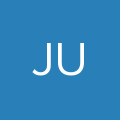


**You**

Secondly, the process of a health app development shows that multidisciplinary (md) and interdisciplinary (id) teamwork is required. We must distinguish between intermittent md, in the sense of juxtaposing disciplinary work and id teamwork with proactive, interactional charac-teristics (29). Nevertheless both, md and id teamwork mean a circular act of science in social care research and is a process with consequently social negotiation. Not only the manage-ment of co-working (because of separate places of work), the formal team meetings and ex-change of information and data, but also the discussions and informal team meetings about preferences, relevancies and disputes must be endure and overcome. Because every scien-tific discipline has a specific domain of its own, i.e., concepts and methodologies, multiple constraints and barriers between different disciplines must be dismantled and integrated (30). Resulting md and id teamwork requires an openness to the knowledge, structures, and prac-tices of the other discipline (31) with a consistent advocacy of the own discipline at the same time. Permanent communication is required as well as compromise solutions (19).

ChatGPT

**ChatGPT**

Secondly, the process of developing a health app underscores the necessity of multidisciplinary (MD) and interdisciplinary (ID) teamwork. It's crucial to differentiate between intermittent MD, characterized by juxtaposed disciplinary work, and ID teamwork, which entails proactive, interactive collaboration (29). However, both MD and ID teamwork entail a circular scientific endeavor in social care research and involve a process of social negotiation. Effective collaboration involves not only managing co-working due to separate work locations, formal team meetings, and the exchange of information and data but also engaging in discussions and informal team meetings to address preferences, relevancies, and disagreements. Given that each scientific discipline possesses its own specific domain, concepts, and methodologies, multiple constraints and barriers between different disciplines must be dismantled and integrated (30). Successful MD and ID teamwork necessitates openness to the knowledge, structures, and practices of other disciplines (31) while consistently advocating for one's own discipline. Continuous communication and compromise solutions are essential requirements for effective collaboration (19).


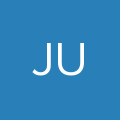


**You**

Thirdly, further generations will use more technical applications for life in general and in a health associated context (32). Hence, it seems essential to investigate and explore the de-velopment of technical instruments, although the actual older generation is not that technical affine like the current generation of adults and youth, but it is expected that the use and ac-ceptance of digital technologies will increase in future (ibid.).

ChatGPT

**ChatGPT**

Thirdly, future generations are expected to rely more heavily on technical applications for various aspects of life, including healthcare (32). Therefore, it is crucial to examine and delve into the development of technical tools, even though the current older generation may not be as technologically savvy as today's adults and youth. However, it is anticipated that the usage and acceptance of digital technologies will grow in the future (ibid.).


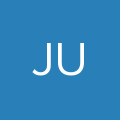


**You**

Additionally, there remains ambiguity surrounding technical solutions (14). The participation of GPs and employees from Alzheimer's Societies provided practical in-sights from healthcare providers engaged in daily interactions with PwD and their caregivers. They shared professional experiences and highlighted daily challenges and needs for im-provement. This collaborative process can enhance the acceptance and utilization of the ap-plication (19). Secondly, the process of developing a health app underscores the necessity of multidiscipli-nary (MD) and interdisciplinary (ID) teamwork. It's crucial to differentiate between intermittent MD, characterized by juxtaposed disciplinary work, and ID teamwork, which entails proactive, interactive collaboration (29). However, both MD and ID teamwork entail a circular scientific endeavor in social care research and involve a process of social negotiation. Effective collab-oration involves not only managing co-working due to separate work locations, formal team meetings, and the exchange of information and data but also engaging in discussions and informal team meetings to address preferences, relevancies, and disagreements. Given that each scientific discipline possesses its own specific domain, concepts, and methodologies, multiple constraints and barriers between different disciplines must be dismantled and inte-grated (30). Successful MD and ID teamwork necessitates openness to the knowledge, struc-tures, and practices of other disciplines (31) while consistently advocating for one's own disci-pline. Continuous communication and compromise solutions are essential requirements for effective collaboration (19). Thirdly, future generations are expected to rely more heavily on technical applications for various aspects of life, including healthcare (32). Therefore, it is crucial to examine and delve into the development of technical tools, even though the current older generation may not be as technologically savvy as today's adults and youth. However, it is anticipated that the usage and acceptance of digital technologies will grow in the future (ibid.).

ChatGPT

**ChatGPT**

Additionally, there remains ambiguity surrounding technical solutions (14).

The participation of GPs and employees from Alzheimer's Societies provided practical insights from healthcare providers engaged in daily interactions with Persons with Dementia (PwD) and their caregivers. They shared professional experiences and highlighted daily challenges and needs for improvement. This collaborative process can enhance the acceptance and utilization of the application (19).

Secondly, the process of developing a health app underscores the necessity of multidisciplinary (MD) and interdisciplinary (ID) teamwork. It's crucial to differentiate between intermittent MD, characterized by juxtaposed disciplinary work, and ID teamwork, which entails proactive, interactive collaboration (29). However, both MD and ID teamwork entail a circular scientific endeavor in social care research and involve a process of social negotiation. Effective collaboration involves not only managing co-working due to separate work locations, formal team meetings, and the exchange of information and data but also engaging in discussions and informal team meetings to address preferences, relevancies, and disagreements. Given that each scientific discipline possesses its own specific domain, concepts, and methodologies, multiple constraints and barriers between different disciplines must be dismantled and integrated (30). Successful MD and ID teamwork necessitates openness to the knowledge, structures, and practices of other disciplines (31) while consistently advocating for one's own discipline. Continuous communication and compromise solutions are essential requirements for effective collaboration (19).

Thirdly, future generations are expected to rely more heavily on technical applications for various aspects of life, including healthcare (32). Therefore, it is crucial to examine and delve into the development of technical tools, even though the current older generation may not be as technologically savvy as today's adults and youth. However, it is anticipated that the usage and acceptance of digital technologies will grow in the future (ibid.).


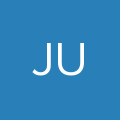


**You**

This paper shows that multiple perspectives like scientific, clinical, and technical are neces-sary and urgently needed to ensure high quality development of supportive healthcare apps. The content and design of such an app for GPs, PwD and their caregivers need to be based on the expertise and experiences of different professional disciplines which are involved in the health care provision and the use of such apps in a real healthcare setting. So, multidiscipli-nary and interdisciplinary work is indispensable in the field of health care supporting apps. Medical disciplines, social and technical science and of course supporting organizations are required to tie relevant knowledge and competences into the design and development of use-ful products, for daily use and active support for healthcare belongings. Besides, such devel-opment processes need a spiral approach and feedback loops as well they do not work with-out professional and emotional critical discussions to find a common ground for taking deci-sions.

ChatGPT

**ChatGPT**

This paper underscores the necessity of multiple perspectives, including scientific, clinical, and technical, to ensure the high-quality development of supportive healthcare apps. The content and design of such apps for General Practitioners (GPs), Persons with Dementia (PwD), and their caregivers must be informed by the expertise and experiences of various professional disciplines involved in healthcare provision and the utilization of such apps in real healthcare settings. Therefore, multidisciplinary and interdisciplinary collaboration is indispensable in the realm of healthcare-supporting apps. Medical disciplines, social sciences, technical expertise, and supporting organizations are all essential to integrate relevant knowledge and competencies into the design and development of useful products, facilitating daily use and active support for healthcare needs. Furthermore, such development processes require a spiral approach and feedback loops, as well as professional and emotional critical discussions, to reach consensus and make informed decisions.


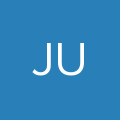


**You**

JS was primary contributor to data analysis, data interpretation and manuscript drafting. SL, JLO, JNVA, RS assisted with the writing and interpretation of findings. JN, and JNVA de-signed and led the DemTab study in which the present study was embedded. All authors con-tributed to the present study and critically revised and approved the final manuscript.

ChatGPT

**ChatGPT**

JS was the primary contributor to data analysis, data interpretation, and manuscript drafting. SL, JLO, JNVA, and RS assisted with writing and interpreting findings. JN and JNVA designed and led the DemTab study in which the present study was embedded. All authors contributed to the present study and critically revised and approved the final manuscript.


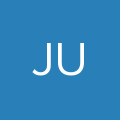


**You**

The authors extend their special appreciation to Sebastian Möller and Adelheid Kuhlmey who supported the application process of the DemTab study and all workshop participants, em-ployees of the day clinic and all study participants. We used the generative AI tool ChatGPT by OpenAI* to correct the English language. The original ChatGPT transcripts are made available as Multimedia Appendix.

ChatGPT

**ChatGPT**

The authors would like to express their special appreciation to Sebastian Möller and Adelheid Kuhlmey for their support in the application process of the DemTab study, as well as to all workshop participants, employees of the day clinic, and all study participants.

We utilized the generative AI tool ChatGPT by OpenAI* to correct the English language. The original ChatGPT transcripts are provided as Multimedia Appendix.

Formularbeginn
